# Supplementary material for: A Comparison of Non-Destructive Visceral Swab and Tissue Biopsy Sampling Methods for Genotyping-by-Sequencing in the Freshwater Mussel Fusconaia askewi
Source: Genes (Basel). 2023 May 30;14(6):1197. doi: 10.3390/genes14061197 (PMC10298480; doi:10.3390/genes14061197)

Table S1. DNA concentrations (ng/ml) as measured with a Qubit system for each sample. Each individual was measured three times (Measure 1, Measure 2, and Measure 3). Also provided are the mean and one standard deviation (SD) for each individual sampled.

		Tissue					Swab				
**Individual number**	**Sample ID**	**Measure 1**	**Measure 2**	**Measure 3**	**Mean**	**SD**	**Measure 1**	**Measure 2**	**Measure 3**	**Mean**	**SD**
1	FUCH-SAB01-01	44.9	48.8	50	**47.9**	**2.7**	30.5	36.3	36.8	**34.5**	**3.5**
2	FUCH-SAB01-02	18.7	22.5	23.6	**21.6**	**2.6**	15.4	17.2	19.2	**17.3**	**1.9**
3	FUCH-SAB01-07	30.4	32.1	32.7	**31.7**	**1.2**	0	0	0.1	**0.02**	**0**
4	FUCH-SAB01-09	12.1	13.6	14.8	**13.5**	**1.4**	24.2	28.9	29.4	**27.5**	**2.9**
5	FUCH-SAB01-16	48.7	52	53	**51.2**	**2.3**	41.1	47.6	49	**45.9**	**4.2**
6	FUCH-SAB01-17	12.3	14.7	15.7	**14.2**	**1.8**	17.8	25.7	26	**23.2**	**4.7**
7	FUCH-SAB01-20	41	43.1	37.6	**40.6**	**2.8**	23.8	21.7	21.9	**22.5**	**1.2**
8	FUCH-SAB01-22	29.1	26.8	27.7	**27.9**	**1.2**	23.9	34.4	35.1	**31.1**	**6.3**
9	FUCH-SAB08-03	48.9	57	60	**55.3**	**5.7**	24.4	26.4	28.4	**26.4**	**2**
10	FUCH-SAB08-08	47.8	48.3	50	**48.7**	**1.2**	29.4	31.2	34.3	**31.6**	**2.5**
11	FUCH-SAB08-13	51.1	53	55.9	**53.3**	**2.4**	50	53	55	**52.7**	**2.5**
12	FUCH-SAB08-15	43.9	49.2	47.3	**46.8**	**2.7**	17.1	20.4	19.9	**19.1**	**1.8**
13	FUCH-SAB08-16	22.9	25.3	26.7	**25**	**1.9**	24.4	28	29.2	**27.2**	**2.5**
14	FUCH-SAB08-20	27.7	30	30.9	**29.5**	**1.7**	27.1	31.9	31.4	**30.1**	**2.6**
				**Mean**	**36.2**				**Mean**	**27.8**	
				**SD**	**14.6**				**SD**	**12.5**	

Table S2. Number of raw reads for each sampling method from each individual. Also provided are the mean and standard deviation (SD) for each individual.

Individual number	Individual	Tissue reads	Swab Reads
1	FUCH-SAB01-01	1,368,358	345,646
2	FUCH-SAB01-02	1,923,997	662,776
3	FUCH-SAB01-07	1,001,351	661
4	FUCH-SAB01-09	1,670,977	553,615
5	FUCH-SAB01-16	1,362,617	441,449
6	FUCH-SAB01-17	1,129,177	247,797
7	FUCH-SAB01-20	1,510,733	317,635
8	FUCH-SAB01-22	1,564,947	375,755
9	FUCH-SAB08-03	1,851,847	807,834
10	FUCH-SAB08-08	1,929,548	820,802
11	FUCH-SAB08-13	1,674,671	670,438
12	FUCH-SAB08-15	1,646,674	631,645
13	FUCH-SAB08-16	1,924,218	737,314
14	FUCH-SAB08-20	1,964,966	652,422
	**Mean**	**1,608,862.93**	**518,984.93**
	**SD**	**307,129.83**	**238,072.90**

Table S3. Median, mean, and standard deviation (SD) of read depth for each sample type when aligned to the tissue-based control reference.

		**Tissue Samples**			**Swab Samples**		
**Individual number**	**Individual**	**Median**	**Mean**	**SD**	**Median**	**Mean**	**SD**
1	FUCH-SAB01-01	3	4.51	6.25	3	5.88	9.59
2	FUCH-SAB01-02	4	6.28	8.60	5	10.95	17.94
3	FUCH-SAB01-07	2	3.35	4.84	0	0.01	0.10
4	FUCH-SAB01-09	4	5.62	8.19	5	8.97	14.32
5	FUCH-SAB01-16	3	4.45	6.57	4	7.47	11.86
6	FUCH-SAB01-17	3	3.81	5.45	2	3.94	6.89
7	FUCH-SAB01-20	3	5.00	7.66	3	5.04	8.17
8	FUCH-SAB01-22	4	5.36	7.59	3	6.44	10.80
9	FUCH-SAB08-03	4	5.97	8.31	7	13.74	22.60
10	FUCH-SAB08-08	5	6.58	9.25	7	14.55	24.34
11	FUCH-SAB08-13	4	5.46	7.45	5	10.70	16.87
12	FUCH-SAB08-15	4	5.21	7.67	5	10.52	17.86
13	FUCH-SAB08-16	4	6.45	9.20	6	12.44	20.08
14	FUCH-SAB08-20	4	6.36	9.00	5	10.77	18.57
	**Mean**	**3.64**	**5.31**		**4.29**	**8.67**	
	**SD**	**0.74**	**1.00**		**1.94**	**4.08**	

Table S4. Median, mean, and standard deviation (SD) of read depth for each sample type when aligned to their respective *de novo* references (e.g., swab samples aligned to swab-only reference).

		**Tissue Samples**			**Swab Samples**		
**Individual number**	**Individual**	**Median**	**Mean**	**SD**	**Median**	**Mean**	**SD**
1	FUCH-SAB01-01	6	10.46	18.46	16	29.72	35.84
2	FUCH-SAB01-02	8	14.99	25.41	30	57.43	72.40
3	FUCH-SAB01-07	4	7.91	14.68	0	0.06	0.37
4	FUCH-SAB01-09	7	13.58	24.77	24	46.70	60.60
5	FUCH-SAB01-16	5	10.21	20.30	20	39.36	49.70
6	FUCH-SAB01-17	5	8.92	16.87	10	18.78	24.50
7	FUCH-SAB01-20	7	12.82	22.04	12	25.55	34.25
8	FUCH-SAB01-22	7	13.11	23.19	18	33.68	40.47
9	FUCH-SAB08-03	7	14.05	25.05	34	73.05	96.86
10	FUCH-SAB08-08	8	15.41	29.12	37	80.09	112.50
11	FUCH-SAB08-13	7	13.16	21.59	27	54.19	66.99
12	FUCH-SAB08-15	6	12.27	22.71	28	53.61	66.79
13	FUCH-SAB08-16	9	15.86	27.13	33	64.92	84.33
14	FUCH-SAB08-20	9	15.67	26.06	28	55.38	73.40
	**Mean**	**6.79**	**12.74**		**22.64**	**45.18**	
	**SD**	**1.48**	**2.53**		**10.50**	**22.04**	

Figure S1: PCA plots for all four datasets: A) swab aligned to swab reference, B) tissue aligned to tissue reference, C) swab aligned to control reference, D) tissue aligned to control reference.

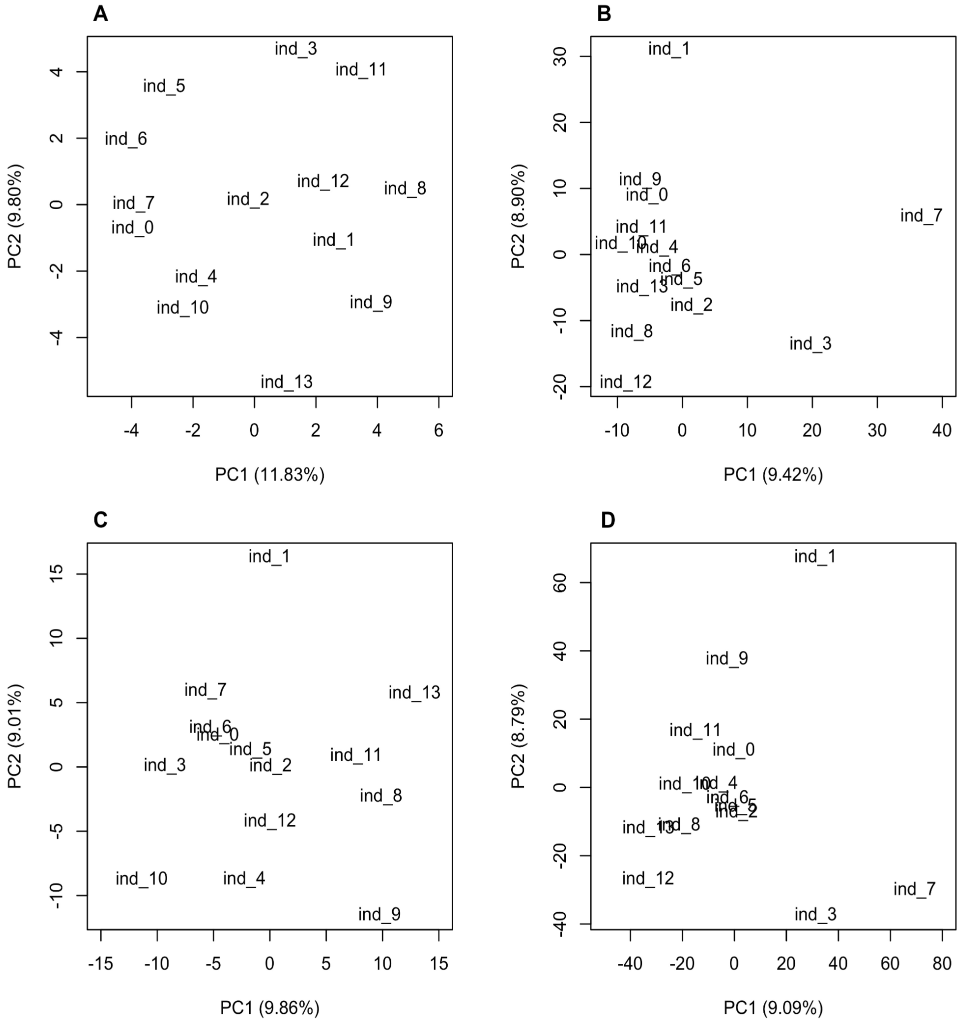

Supplement: Supplementary file 1 [file genes-14-01197-s001.zip › genes-2406378-supplementary.pdf]
